# Metabolic interaction: tumor-derived lactate inhibiting CD8^+^ T cell cytotoxicity in a novel route

**DOI:** 10.1038/s41392-023-01320-y

**Published:** 2023-02-03

**Authors:** Ke Wang, Yong Zhang, Zhi-Nan Chen

**Affiliations:** 1 National Translational Science Center for Molecular Medicine & Department of Cell Biology, Fourth Military Medical University, Xi’an, 710032 China; 2 Department of Pulmonary and Critical Care Medicine, the First Affiliated Hospital of Fourth Military Medical University, Xi’an, 710032 China

**Keywords:** Cancer metabolism, Cancer microenvironment

Recently, an interesting study published in *Cell Metabolism* discovered a novel metabolic interaction between tumors and T cells, identifying that tumor-derived lactate inhibited CD8^+^ T cell cytotoxicity by inducing a switch of pyruvate utilization from pyruvate carboxylase (PC) to pyruvate dehydrogenase (PDH), and PDH inhibition facilitated PC activity and T cell cytotoxicity through increasing succinate secretion, in turn leading to synergistic effects on tumor therapy with immunotherapy, which provides a promising model for tumor treatment targeting T cell metabolism.^1^

Metabolic reprogramming, a hallmark of cancer, plays a critical role on tumor progression. Cancer cells autonomously alter their metabolic patterns to meet increased bioenergy and biosynthetic demands, and affect the fate of other cells in the tumor microenvironment (TME) to achieve the goal of tumor progression, including tumor-associated fibroblasts, endothelial cells, and immune cells. In particular, CD8^+^ T cells, as an important anti-tumor pioneer, are commonly affected by tumor metabolic microenvironment, leading to impaired cell proliferation, activation, and survival.^2^ Therefore, the investigation of metabolic interactions between tumor and CD8^+^ T cells in TME contributes to understand the pathophysiology and clinical metabolic phenotype of tumors, which is expected to provide a new theoretical basis for cancer treatment strategies targeting metabolic pathways.

In this study, Elia et al. found that nutrient conditions mimicking the TME impaired CD8^+^ T cell cytotoxicity. In order to assess the metabolic changes of T cell, they developed a technique for rapid isolation of T cells from co-cultures and observed that nutrient composition derived from tumor cells could inhibit PC activity, leading to T cell metabolic reprogramming. Further study confirmed tumor-derived lactate was the specific metabolite, which decreased PC activity and CD8^+^ T cell anti-tumor responses.

Lactate is reported to be one of the most prominent metabolites of the TME. Tumor cells employ lactate as energy source to sustain metabolic demands, while immune cells perceive lactate as potential signal to promote the formation of immunosuppressive microenvironment. The Warburg effect (aerobic glycolysis) of cancer cells contributes to the accumulation of lactate, which is associated with the activity of several major glycolytic enzymes, especially lactate dehydrogenase A (LDHA). LDHA-associated lactate production and acidification diminish the levels of nuclear factor of activated T cells and T and NK cell activation, leading to immune evasion.^3^ Meanwhile, high level of lactate also promotes PD-1 expression in regulatory T cells to sustain its immunosuppressive function.^4^ Mechanically, this work found tumor-derived lactate as an inhibitor of CD8^+^ T cell cytotoxicity, inducing a switch of pyruvate utilization from PC to PDH and altering T cell metabolic reprogramming. Glycolysis-derived pyruvate provides the TCA cycle with acetyl-CoA via PDH route and oxaloacetate via PC route. The dilemma in metabolic selection of pyruvate depends on relative activation between PDH and PC, in which elevated PC activity may be overlapped with PDH inhibition. In this study, the authors validated the hypothesis that PDH inhibition increased pyruvate metabolism for PC activity in a lactate-dependent manner, which facilitated succinate secretion. Succinate, as a key intermediate of TCA cycle, is closely associated with the release of inflammatory factors by binding its specific G protein-coupled receptor succinate receptor 1 (SUCNR1). Subsequently, Elia et al. found that the increase in succinate resulting from PDH inhibition activated SUCNR1 and enhanced CD8^+^ T cell-specific killing and IFNγ production, while lactate-induced cell cytotoxicity impairment was not rescued in CD8^+^ T cells with SUCNR1 knock-down. Meanwhile, inhibition of PDH in vivo promoted anti-tumor immunity and exhibited synergistic effects on tumor therapy with immunotherapy. These findings bring a promising future for cancer therapy by targeting PDH. However, the potential challenges of targeting PDH still need to be explored, including the evaluation of lactate accumulation degree, the effect on the metabolic status of uncorrelated cells, and the regulation of glucose homeostasis.

**Fig. 1:**
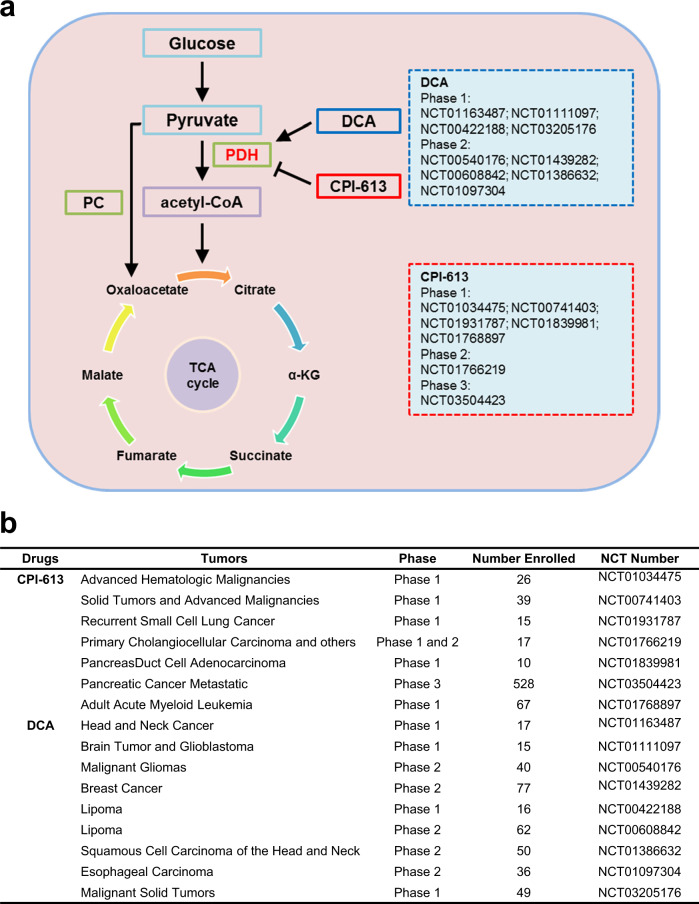
Drugs targeting PDH in clinical trials. **a** In the absence of lactate, pyruvate may step into PC-mediated metabolic process with accumulated oxaloacetate, which can be converted into PDH pathway by additional lactate from TME. These metabolic outcomes from both enzyme catalytic pathways participate TCA cycles to support energy supplement. DCA and CPI-613, as small molecule targeted drugs, indirectly present positive and negative effects on PDH, respectively. **b** Several completed clinical trials within pan cancers are listed with clinical phases and NCT numbers

Of note, the persistent efforts for targeting potential metabolic vulnerabilities have acquired several phased achievements. A series of agonists or inhibitors targeting key metabolic enzymes in tumor cells have been under clinical trials, including those in TCA cycle.^5^ For instance, CPI-613, a lipoamide mimic reducing PDH activity used in this study, weakened the glucose uptake of tumor cells significantly and exerted non-toxic effects to normal counterparts in preclinical study, which were also verified in breast and hematological malignancies in clinical trials. In a single center and open labeled phase I trial, CPI-613 (Devimistat, *Rafael*) combined with modified FOLFIRINOX (mFFX) exhibited an inspiring outcomes with a 61% objective response rate including a 17% complete response rate in pancreatic cancer patients. Correspondingly, as an agonist of PDH used to treat lactic acidosis, dichloroacetate (DCA) transformed glycolytic flux into oxidative metabolism in certain types of tumors, indicating its potential clinical applications in restricting energy supplement of tumors despite limited cytotoxicity (Fig. 1). Additionally, mutations within separate isoforms of NADP-dependent isocitrate dehydrogenase (mIDH1 and mIDH2), which blocked the catalysis from isocitrate to α-ketoglutarate (α-KG) within cytoplasm and mitochondrial respectively and produced intermediate metabolites to inhibit competitively with α-KG-dependent enzymes, could be antagonized by inhibitors of mIDH1 and mIDH2 (AG-120, AG-881, and AG-221) against solid tumors, refractory AML, and myelodysplastic syndrome. As a FDA approved drug for the treatment of adult patients with unresectable locally advanced or metastatic hepatocellular IDH1 mutated cholangiocarcinoma in 2021, AG-120 (Ivosidenib, *Tibsovo*) conferred them with a median follow-up of 6.9 months and median overall survival (OS) of 10.3 months, compared with that of 7.5 months in placebo arms. AG-221 (Enasidenib, *Idhifa*) was also approved for relapsed or refractory AML by FDA in 2017 with overall response rate of 40.3%, median response duration of 5.8 months, and OS of 19.7 months. AG-881 (Vorasidenib, *Servier*), as a first-in-class, brain-penetrant, dual inhibitor of the mutant IDH1 and mutant IDH2 enzymes, showed a favorable safety profile in the glioma cohort with median progression-free survival of 36.8 months [95% confidence interval (CI), 11.2–40.8] for patients with nonenhancing glioma and 3.6 months [95% CI, 1.8–6.5] for patients with enhancing glioma in a multicenter Phase I study. Unfortunately, these drugs were usually evaluated as single agent to act on different tumor cells, but failed to examine their properties in igniting T cell cytotoxicity and their synergism with anti-tumor immunotherapy drugs in vitro and in vivo, which deserved further investigations in fundamental research. In correspondence with ongoing clinical trials of TCA metabolic enzymes, targeting catalytic pathways of pyruvate in tumor-infiltrated T cells may be a potential strategy to optimize anti-tumor efficacy, which still needs controlled clinical trials with large samples in multi centers.

Briefly, this study uncovers a brand new insight into the impact of TME on T cell metabolic reprogramming, which innovatively illustrates the relationship between pyruvate shunt metabolism and CD8^+^ T cell function from the perspective of metabolic microenvironment, and provides the therapeutic potential of targeting T cell metabolism to strengthen cytotoxicity in tumor immunotherapy. As stated by the authors, although due to technical challenges, validation of these findings in clinical samples is still necessary. Meanwhile, it is also required to investigate the detailed mechanism of balancing pyruvate utilization between PC and PDH routes.
